# Bone Histology Reveals a High Environmental and Metabolic Plasticity as a Successful Evolutionary Strategy in a Long-Lived Homeostatic Triassic Temnospondyl

**DOI:** 10.1007/s11692-013-9238-3

**Published:** 2013-05-22

**Authors:** S. Sanchez, R. R. Schoch

**Affiliations:** 1 Subdepartment of Evolutionary Organismal Biology, Department of Physiology and Developmental Biology, Evolutionary Biology Centre, Uppsala University, Norbyvägen 18A, 752 36 Uppsala, Sweden; 2 Staatliches Museum für Naturkunde, Rosenstein 1, 70191 Stuttgart, Germany

**Keywords:** Morphological stasis, Developmental plasticity, Reaction norm, Environmental canalization

## Abstract

Evolutionary stasis (long-term stability of morphology in an evolving lineage) is a pattern for which explanations are usually elusive. The Triassic tetrapod *Gerrothorax pulcherrimus*, a gill-bearing temnospondyl, survived for 35 million years in the Germanic Basin of Central Europe persisting throughout the dinosaur-dominated Late Triassic period. This evolutionary stasis coincides with the occurrence of this species in a wide range of habitats and environmental conditions. By the combination of palaeoecological and palaeohistological analyses, we found great ecological flexibility in *G. pulcherrimus* and present substantial evidence of developmental and metabolic plasticity despite the morphological stasis. We conclude that *G. pulcherrimus* could show the capacity to settle in water bodies too harsh or unpredictable for most other tetrapods. This would have been made possible by a unique life history strategy that involved a wide reaction norm, permitting adjustment to fluctuating conditions such as salinity and level of nutrients. Growth rate, duration of juvenile period, age at maturity, and life span were all subject to broad variation within specimens of *G. pulcherrimus* in one single lake and in between different lakes. In addition to providing a better understanding of fossil ecosystems, this study shows the potential of such a methodology to encourage palaeobiologists and evolutionary biologists to consider the mechanisms of variation in extant and fossil organisms by using a similar time-scope reference.

## Introduction

Understanding the processes that influence the survival and extinction of evolutionary lineages is a major task of (palaeo-) biology. Many studies revealing the causes of mass extinction events have been proposed not only by palaeontologists (e.g., Benton 1995; McElwain and Punyasena 2007; Ruta and Benton 2008; Smith and Ward 2001; Tanner et al. 2004) but also by ecologists (e.g., Mayhew et al. 2008; Thomas et al. 2004). Conversely, long-term survival of taxa has fascinated evolutionists for decades (e.g., Bocxlaer et al. 2008; Debat et al. 2000; Eldredge and Gould 1972; Eldredge et al. 2005; Flatt 2005; Futuyma 2010; Hansen and Houle 2004; Kemp 1999; Stenseth and Smith 1984; Waddington 1957), but the causes for increased evolutionary stability and stasis usually remain elusive (Debat and David 2001; Futuyma 2010; Gould 2002). Most interesting are the cases for which the phenotypic homeostasis can be analysed in environmental, developmental and genetic contexts. Firstly, organism-wide morphological stasis was thought to reflect canalisation and developmental homeostasis (Eldredge and Gould 1972; Waddington 1957), but many studies have since shown that genotype–phenotype, development, and environment relationships are far more complex (e.g., Charlesworth et al. 1982; Debat and David 2001; Debat et al. 2000; Futuyma 2010) and can lead to unsuspected variability despite clear morphological constancy in a single lineage (Flatt 2005). Checking therefore the genetic, developmental, and environmental variability in homeostatic taxa is of major interest for a better understanding of evolutionary stasis. From a time-scale point of view, the most obvious study cases of evolutionary stasis usually come from the fossil record. Because it is mostly impossible to get access to the genetic pool in fossil samples, the assessment of the potential genetic variability in a fossil lineage can only be supplemented by the measurement of the palaeobiological/palaeophysiological plasticity rendered possible through an analysis of the bone histological record. Based on ontogenetic, metabolic, and palaeoecological data, plausible scenarios can then be proposed for understanding the long-term evolutionary success of fossil populations and taxa.

Here, we report on such a case from a long-extinct tetrapod, the temnospondyl *Gerrothorax pulcherrimus*. This plagiosaurid, a flat-bodied aquatic form with extensive dermal armour, is interpreted as a suction feeder at the bottom of Triassic lakes and streams (Hellrung 2003; Jenkins et al. 2008). *Gerrothorax* was an obligatorily aquatic taxon, which retained lateral line organs and gills in adults (Schoch and Witzmann 2011). Recently, Schoch and Witzmann (2012) studied the ontogeny and stratigraphic distribution of *G. pulcherrimus*, finding that: (1) the species existed for at least 35 million years (My) in the Germanic Basin; (2) its general morphology remained practically unchanged through this time span; and (3) it occurred in a wide range of palaeoenvironments. This pattern is exceptional among fossil amphibians in two respects: first, the extent and duration of evolutionary stasis; and second, the apparent ecological flexibility through time and space. The causes of both patterns form the focus of the present analysis.

The objective of the current study first is to analyze growth rates, individual ages, timing of sexual maturity, and metabolic traits in two samples of *G. pulcherrimus* from two different German localities. Secondary, these data will be compared to another group from one of these localities (*Plagiosuchus* *pustuliferus*) and then integrated with rich data on the palaeoenvironments. The final aim is to match the inferred life history traits with environmental and ecological parameters, in order to understand the evolutionary strategy of *Gerrothorax*.

## Materials and Methods

The material studied here was collected in two different German localities: Kupferzell and Vellberg. The time span covered by these localities is maximally a few ten thousand years. The existence time for each lake was much less, supposedly a few hundred years only.

The Kupferzell locality is constituted of a basal green calcareous mudstone horizon (i.e., the lowest stratigraphical layer, which is 20 cm thick, and interpreted as a lake deposit; Schoch and Witzmann 2012) in southern Germany (Baden-Württemberg). It is a now-overbuilt road-cut. The excavated area ranged over 1,000 m^2^, and the collected material amounts to more than 30,000 bones (Wild 1980). The Kupferzell locality comprises other stratigraphic layers but 70–80 % of the total amount of *Gerrothorax pulcherrimus* come from this rich green calcareous mudstone horizon and is accompanied by bones of the five-meter long *Mastodonsaurus giganteus*; only very few, fragmentary finds of other tetrapods were collected in this bed (*Kupferzellia*, *Plagiosuchus*, *Batrachotomus*).

The Vellberg locality is a limestone quarry that has been operating for 60 years. The lower Keuper section has been collected for at least 30 years, yielding rich vertebrate finds in various beds. The richest bed, a grey mudstone bed that formed in a freshwater lake, preserves a temnospondyl fauna consisting of *Mastodonsaurus*, *Kupferzellia*, *Trematolestes*, *Callistomordax*, and *Gerrothorax*.

**Fig. 1:**
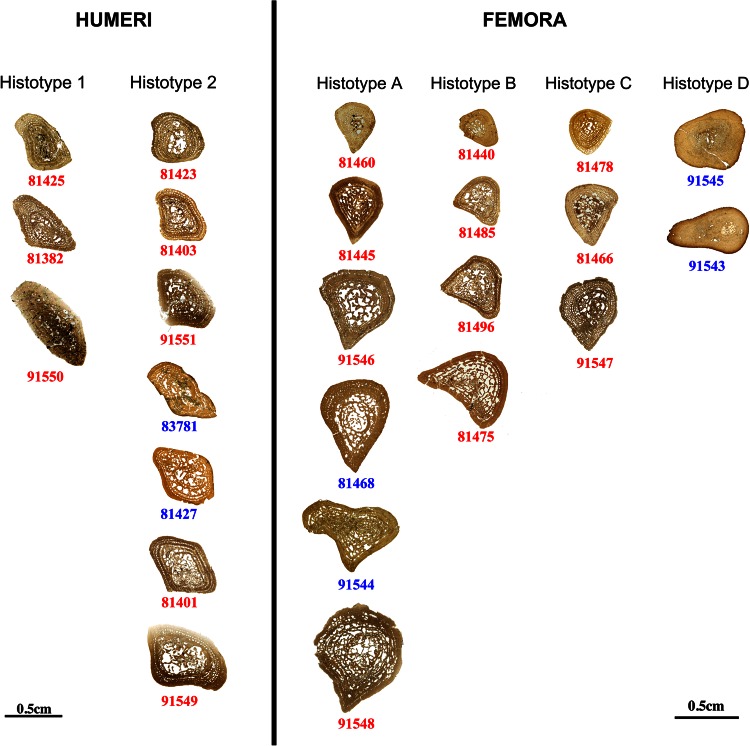
Samples studied. Ontogenetic series of humeri and femora (midshaft thin sections) of *Gerrothorax pulcherrimus* showing the different histotypes. Specimen numbers written in *red* refer to specimens coming from the Kupferzell locality and specimen numbers written in *blue* refer to specimens coming from the Vellberg locality

The studied limb bones are assignable to *G. pulcherrimus*, the most abundant tetrapod sampled at Kupferzell and Vellberg (Hellrung 2003; Schoch and Witzmann 2012), and *Plagiosuchus pustuliferus* from the same Kupferzell bed (green calcareous mudstone layer). Regarding *G. pulcherrimus*, two sets of humeri (8 for Kupferzell and 2 for Vellberg) and two sets of femora (11 for Kupferzell and 4 for Vellberg) are studied in this paper (Fig. 1). In total, twenty-five long bones were sectioned at mid-shaft. Regarding *P. pustuliferus*, four long bones (2 femora and 2 humeri) were sectioned for comparison. All elements sectioned and studied here were compared in detail to those of articulated specimens from the same localities and horizons. Taxonomical confusion can be ruled out because the anatomy of *G. pulcherrimus* is highly distinctive (Hellrung 2003); even when compared to the close relative *P. pustuliferus* (Damiani et al. 2009). Both the humerus and femur of *Plagiosuchus* are readily distinguishable from those of *Gerrothorax* by their much stouter appearance, even at small ontogenetic stages, and the characteristic coarse surface at the articular ends of the long bones. In *Plagiosuchus*, the humerus lacks a supinator process, and the femur has a much shallower crest than in *Gerrothorax* (Hellrung 2003). A further consistent difference is that the limb elements of *Plagiosuchus* have a more rudimentary appearance than those of *Gerrothorax*, particularly in the extent of bone in the proximal and distal ends of the humerus; only the largest specimens of *Plagiosuchus* reach the morphological condition that is typical of *Gerrothorax*.

The current study focusses on limb bone histology. This latter is usually very informative, especially within the framework of growth series. The long-bone cortex is centrifugally appositionally deposited following the regularity of yearly biological cycles (Castanet 1985; Castanet et al. 1993). The nature of the successive bone deposits therefore reflects the global metabolic activity of an organism (Castanet and Baez 1991; Montes et al. 2007) and any disturbances that could have occurred during its development (Alcobendas and Castanet 2000; Caetano and Castanet 1993; Sanchez et al. 2010b; Steyer et al. 2004).

Because stylopodial bones are informative archives for skeletochronological studies in small-to-medium sized temnospondyls (Sanchez et al. 2010b), we decided to focus on the humeral and femoral midshafts. This is the ossification centre, i.e. the region where the development of the bone commences (Francillon-Vieillot et al. 1990). Long bones were embedded in a polyester resin and then sectioned. Thin sections were observed under natural and polarized light through an optical microscope (Nikon Eclipse 80i). Assessment of age and ontogenetic staging is based on skeletochronology (Castanet 1994; Castanet et al. 1993) and (in some cases) retrocalculation (Castanet et al. 2003).

Bone palaeohistology, as a complementary tool to anatomical and environmental analyses, can provide further information about the palaeobiology and palaeophysiology of extinct taxa (Castanet et al. 1993, 2003; Ricqlès et al. 2004; Konietzko-Meier and Klein 2013; Sanchez et al. 2010a, 2010b; Steyer et al. 2004). In order to avoid any misinterpretation of the bone microstructure variation, which could be related to a phylogenetical signal (Cubo et al. 2005), we decided to focus our study on several specimens of different ontogenetic stages belonging to a single plagiosaurid species, *G.* *pulcherrimus* (the comparison with *P. pustuliferus* only being made as a reference for evaluating *Gerrothorax*’s reaction norm). In order to set our hypotheses in an evolutionary framework, the fossil bone samples were extracted from sedimentary beds representing different palaeoecological niches of varied ages.

## Results

### Limb-Bone Histology of *Gerrothorax pulcherrimus*

Our analysis of the thin sections revealed clear-cut clusters of histological patterns that we define as “histotypes” (Fig. 1): two humeral histotypes and four femoral histotypes. They are discriminated on the basis of differential: cortical compactness; vascular mesh organisations, densities and canal size; remodelling stage; quantities of calcified cartilage; patterns of lines of arrested growth; bone matrix types; bone-cell distributions, size and shape. All these histological parameters are significantly related to metabolic, physiological and biological specific functions (e.g., blood supply, growth speed; see explanations in the discussion part). Because the sectioned material consists of isolated bones, we describe these histotypes for humeri and femora separately. Humeral histotypes were therefore numbered as 1 and 2, femoral histotypes as A, B, C and D.

### Histotype 1 (Figs. 1, 2)

#### Humerus SMNS 81425—Kupferzell Locality

**Fig. 2:**
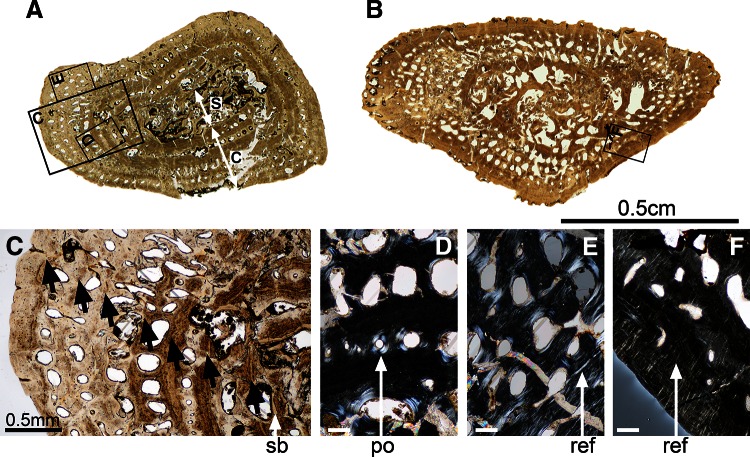
Histotype 1—humerus, *Gerrothorax pulcherrimus*. Mid-shaft transversal thin section made in the humerus 81425 (**a**) show a rather compact cortex (c, **a**) delimited from a loose central spongiosa (s, **a**). In the humerus 81382 (**b**), the spongiosa greatly extends towards the external surface of the bone. This spongiosa is already obviously remodelled, as suggested by the presence of secondary-bone (sb, **c**) deposit in the youngest specimen 81425. Numerous primary osteons (po, **d**) are longitudinally oriented and concentrically aligned in both specimens. Seven lines of arrested growth (*black arrows*, **c**) regularly cross the mid shaft of this specimen and get closer to each other towards the periphery. Radial extrinsic fibres (ref, **e**–**f**) can be observed in both specimens (**e**–**f**). *Scale bars* for the images **d**, **e** and **f**: 0.1 mm. Photos **a**, **b**, **c** taken under natural light; photos **d**, **e**, **f** taken under polarized light

The mid-shaft section of the humerus of this specimen is elongated and triangular (Fig. 2a). It has a relatively well-preserved peripheral primary cortex (1.8 mm thick in average; c, Fig. 2a) whereas the inner central spongiosa of the section is crushed (s, Fig. 2a). This indicates that the medullary cavity must have been substantially eroded and the trabecular bony structure of the medulla relatively weak. The inner part of the primary cortical surface is not visible. This completes the previous observation: the inner part of this cortex must have been eroded as well in continuation of the medullary cavity, forming an extended spongiosa. The structure of the trabeculae reveals that the uncrushed part of the spongiosa is mainly secondary (sb, Fig. 2c, d). There is no remnant of calcified cartilage. The remaining cortical bone is substantially remodelled, including numerous primary osteons (po, Fig. 2d, e). The canal diameters of these osteons range between 85 and 125 μm on average. These osteons are longitudinally oriented, i.e. their long axis parallels the bone long axis, and they are concentrically aligned (Fig. 2d, e). Up to six series of osteon rows can be counted. The bone matrix forms a fibro-lamellar system. Radial extrinsic fibres are present in certain areas, especially the periphery of the cortex (ref, Fig. 2e). Seven lines of arrested growth (LAGs) were identified (black arrows, Fig. 2c): in the periphery, the distance between each LAG is around 0.25 mm, contrasting with the distance of 0.41 mm in the inner region. The osteocyte lacunae in the primary tissue are round, small (6 μm) and evenly distributed. In the secondary bone deposits, bone cells are bigger (20.5 μm long in average), flattened and aligned along the vascular-canal surface.

#### Humerus SMNS 81382—Kupferzell Locality

This whole section is relatively well preserved in three dimensions, except for crushing in the central most part (Fig. 2b). The cross-section is elongated and triangular. The outer surface is irregular and damaged, indicating weathering or transport before final burial, which are usual phenomena at Kupferzell. As preserved, the average cortical thickness is 2.4 mm. The vascular canals within the primary bone matrix of the cortex are more eroded than those of SMNS 81425 (Fig. 2a, b). By average, their diameters are about 145 μm. They form large primary osteons, that overprint the original organization in rows. The osteons are oriented longitudinally. The primary bone matrix is fibrous, and the spongiosa is made of remodelled trabeculae that are oriented in all directions. There is no remnant of calcified cartilage (Sanchez et al. 2010a). The primary bone tissue includes radial extrinsic fibres (Fig. 2f), which are more numerous in the external half of the cortex. Two LAGs were identified in the peripheral cortex, but no LAG pattern can be obviously recognized from the lamellae in the innermost part of the cortex. The distance between the peripheral LAGs is 0.20 mm, and the size of osteocyte lacunae measures 7 μm in the primary tissue. They are round in most cases even if few lacunae are slightly flattened. Osteocyte lacunae in the secondary bone deposits are 15 μm long and 7 μm wide. They are aligned in circles around the blood vessels.

#### Humerus SMNS 91550—Kupferzell Locality

The mid-shaft section of this bone is elongated but crushed in its central part. The medullary cavity and the innermost part of the primary cortical bone must have therefore been substantially eroded, thereby forming an extended secondary spongiosa. There is no remnant of calcified cartilage. The remaining cortical bone is substantially remodelled, including numerous primary osteons. The canal diameters of these osteons can reach a diameter of 165 μm on average. They are longitudinal or radial and concentrically aligned. Only two rows of vascular canals are still embedded in the primary cortex. The rest has been integrated into the spongiosa through erosion process. The bone matrix forms a fibro-lamellar system. There is no clear LAG pattern. The osteocyte lacunae in the primary tissue are round, small (7 μm) and evenly distributed. In the secondary bone deposits, bone cells are bigger (14–21 μm long), flattened and aligned along the vascular-canal or erosion-bay surfaces.

### Histotype 2 (Figs. 1, 3)

#### Humerus SMNS 81423—Kupferzell Locality

**Fig. 3:**
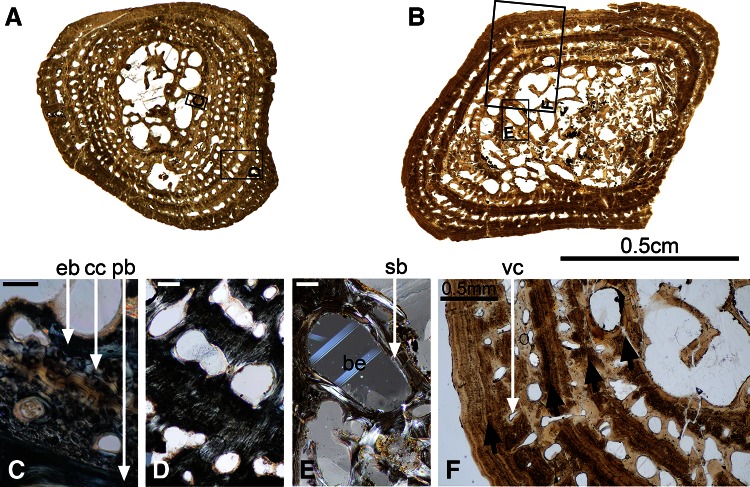
Histotype 2—humerus, *Gerrothorax pulcherrimus*. Mid-shaft transversal thin sections made in the humerus 81423 (**a**) and the humerus 81401 (**b**) show that the erosion process is very active during the ontogeny. Some remnants of calcified cartilage (cc, **c**), present in the youngest individual 81423 between the endosteal bone deposit (eb, **c**) and the primary bone cortex (pb, **c**), are not observable anymore in the biggest and oldest specimen 81401. The cortex of both specimens is made of fibro-lamellar bone, i.e. fibrous primary bone pierced by primary osteons (**d**). In specimen 81401, numerous bays of erosion (be, **e**) appear, due to the remodelling of the cortex. These bays are surrounded by a lamellar secondary bone deposit (sb, **e**). Four lines of arrested growth (*black arrows*, **f**) are visible in periphery of the biggest specimen 81401. They alternate with rows of primary osteons and vacular canals (vc, **f**). *Scale bars* for the images **c**, **d** and **e**: 0.1 mm. Photos **a**, **b**, **f** taken under natural light; photos **c**, **d**, **e** taken under polarized light

This section is of rounded triangular shape and completely preserved (Fig. 3a). The cortical bone is 2 mm thick. The medullary cavity is oval (longest diameter: 3 mm; smallest diameter: 2.1 mm) and crossed by several endosteo-endochondral trabeculae, oriented in all directions. Some remnants of calcified cartilage (cc, Fig. 3c) are still present, both within the medullary trabeculae and between the primary cortical bone (pb, Fig. 3c) and the endosteal bone (eb, Fig. 3c). The cortical bone consists of fibro-lamellar tissue (Fig. 3d) (Francillon-Vieillot et al. 1990). The vascular canals (in the periphery) and the primary osteons are longitudinal (Fig. 3a, d). The lumina of these osteons range between 45 and 130 μm. The vascular mesh is poorly organized in the innermost part of the cortex, but the osteons and canals tend to be arranged in five concentric rows towards the periphery (Fig. 3a). Extrinsic fibres, located in the outermost half of the cortex, are radially oriented. Four LAGs were identified, having an average distance of 0.41 mm between each other. In the primary tissue, the size of the bone-cell lacunae varies between 12.5 (in the innermost central region of the section) and 8 μm (towards the periphery). The lacunae not only decrease in size but also in number towards the periphery. Osteocyte lacunae are rounded and distributed in a random fashion. The flattened lacunae in the secondary bone deposits are 16.5 μm long. Their long axes parallel the bone surface.

#### Humerus SMNS 81403—Kupferzell Locality

This section is triangular, but slightly crushed in its central part. Nevertheless, some medullary trabeculae are still preserved. The outline of the medullary cavity is also triangular, and the thickness of the cortex is relatively constant (2.2 mm in average). The cortex consists of a fibro-lamellar tissue, and the lumina of primary osteons fall into the range of 130–170 μm. The cortical compactness has been significantly reduced by remodelling. The most peripheral osteons are aligned in rows, but towards the centre, the alignment of the vascular canals has been affected by erosion. Most of the osteons are still longitudinally oriented. There are very few remnants of calcified cartilage, located between the endosteal deposit and the primary cortical bone. Several extrinsic fibres are arranged radially. The medullary trabeculae are completely ossified and most of them bear a thin secondary bone deposit. These trabeculae lack any preferential orientation. Three LAGs were identified, located at 0.43 mm from each other. In the primary tissue, the innermost cells are larger (8 μm) than the peripheral ones (6 μm). The peripheral lacunae are gently flattened, but again without consistent orientation. They are less numerous than in 81423. In the secondary bone deposit, few but large (12.5 μm) and flattened cellular lacunae are present, aligned with the surface of the vascular lumen.

#### Humerus SMNS 91551—Kupferzell Locality

This section is triangular and exceptionally well preserved. The cortical thickness varies between 1.5 and 3 mm. The medullary cavity is relatively round with an averaging diameter of 1.5 mm. It is crossed by several endosteo-endochondral trabeculae, oriented in all directions. Some remnants of calcified cartilage can still be observed between the primary cortical bone and the endosteal bone. The cortical bone consists of fibro-lamellar tissue. The vascular canals and the primary osteons in the cortex are all longitudinal. The lumina of these osteons range between 55 and 220 μm. The vascular mesh is organized into six successive concentric rows. Four LAGs were identified, having an average distance of 0.39 mm between each other. The size of the bone-cell lacunae in the primary tissue varies between 14 (in the innermost central region of the section) and 7 μm (towards the periphery). The lacunae decrease in size and number towards the periphery. In the secondary bone deposits, bone cells are largely flattened and measure 21 μm long. Their long axes parallel the bone surface.

#### Humerus SMNS 83781—Vellberg Locality

Due to crushing of the spongiosa, this section is flattened. Very few trabeculae are still in place. However, more than 1 mm of surrounding cortex is preserved. It is fibro-lamellar and pierced with concentric rows of primary osteons (lumina: 90 μm in average). The osteons are all aligned in rows. Most of the osteons are longitudinally oriented. There is still some calcified cartilage preserved in large quantity. Six LAGs were identified, showing a progressive transition from a cyclical deposition of 0.40–0.25 mm. In the primary bone of the cortex, the cell lacunae vary from 10 to 7 μm towards the external bone surface. The peripheral lacunae are gently flattened, but without preferential orientation.

#### Humerus SMNS 81427—Vellberg Locality

This section is square-shaped and relatively well preserved in 3D. The margin of the medullary cavity is not visible anymore because of erosion process. The large spongiosa is surrounded by less than 1 mm of compact cortical bone. The cortex contains some primary osteons (lumen diameter averaging 135 μm) and a few vascular canals. Some large bays of erosion remodelled the organisation of the central spongiosa and appear in between rows of cortical primary osteons. Most of them are covered with a thin layer of secondary bone deposit, as are the medullary trabeculae. There is no visible remnant of calcified cartilage. The primary bone tissue is fibrous. Only one LAG could be identified in the periphery of the cortex. Cell lacunae are round and measure 7 μm in the remaining primary bone. They are few in number but evenly distributed. The osteocytes in the secondary bone tissue are larger (14 μm long), flattened and aligned in parallel to the lamellar bone.

#### Humerus SMNS 81401—Kupferzell Locality

This section is square-shaped (Fig. 3b), with the central part eroded and crushed. The margin of the medullary cavity is not preserved (Fig. 3b). Compactness increases towards the bone surface (Fig. 3b). The cortex contains some primary osteons and a few vascular canals (vc, Fig. 3f), the latter being located in the periphery (lumen diameter ranging between 110 and 160 μm). In certain places, erosion has destroyed the boundary between two lumina, with two bays falling within one lacuna. Remodelling was more extensive in the centre of the cortex, resulting in large bays of erosion (be, Fig. 3e) that are covered with a thin layer of secondary bone deposition (sb, Fig. 3e). The central trabeculae are very delicate and entirely formed by secondary bone tissue. Four consecutive rows of vascular canals and osteons are present in the most peripheral region of the cortex (Fig. 3f). In between these rows, a significant amount of bone remains unaltered by erosion. The primary bone tissue is fibrous. Four LAGs (black arrows, Fig. 3f) were identified in the periphery of the cortex (average distance between each other: 0.40 mm). Cell size ranges from 8 μm in the central region to 4 μm in the periphery of the section, with most of the bone cells being round. They are few in number but evenly distributed. The osteocytes in the secondary bone tissue were larger (16.5 μm long), flattened and aligned in parallel to the lamellar bone.

#### Humerus SMNS 91549—Kupferzell Locality

This section is slightly more triangular than 81401 and almost fully preserved in 3D. Because of intense erosion, the outline of the medullary cavity is not visible anymore. The spongiosa has spread far into the cortex, only leaving an outer compact layer of 1.5 mm thick. The cortex consists of a fibro-lamellar tissue, and the lumina of primary osteons are 150 μm in diameter. The osteons are aligned in rows. Most of the osteons are still longitudinally oriented. There are very few remnants of calcified cartilage, located between the endosteal deposit and the primary cortical bone. The medullary trabeculae are completely covered with a thin secondary bone deposit. These trabeculae lack any preferential orientation. Five LAGs were identified, distant of 0.23 mm from each other in the peripheral region of the compact cortical bone. In the remaining primary bone deposit, bone cell lacunae are gently flattened and measure 9 μm long. They show no preferential orientation. In the secondary bone deposit, they are greatly flattened and aligned with the endosteal surfaces. They are 24 μm long.

### Histotype A (Figs 1, 4)

#### Femur SMNS 81460—Kupferzell Locality

**Fig. 4:**
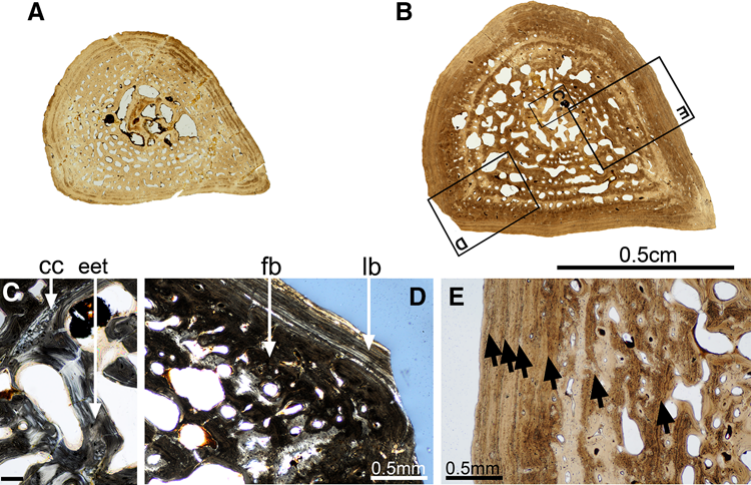
Histotype A—femur, *Gerrothorax pulcherrimus*. Mid-shaft transversal thin sections made in the femur 81460 (**a**) and the femur 81445 (**b**) show a clear distinction between the cortex and the medullary cavity. Despite the visible growth slow-down, revealed by the peripheral tightening of the lines of arrested growth (*black arrows*, **e**) and the shift from primary fibrous bone (fb, **d**) to lamellar bone (lb, **d**), the oldest specimen 81445 still shows some remnants of calcified cartilage (cc, **c**) within the endosteo-endochondral trabeculae (eet, **c**). *Scale bar* for the image **c**: 0.1 mm. Photos **a**, **b**, **e** taken under natural light; photos **c**, **d** taken under polarized light

This section is drop-shaped (Fig. 4a), with the sharp angle formed by the femoral crest. The mid-shaft is preserved in 3D. The medullary cavity and cortex are readily distinguished. The outline of the medullary cavity parallels the outline of the bone (longest diameter: 2.4 mm). The cortex measures 1.8 mm in average and is relatively compact despite a dense vascular mesh. Most of the vascular network is composed of primary osteons (lumen diameters ranging between 70 and 105 μm). There are also a few simple vascular canals. The vascular lacunae are generally small but larger in the innermost region of the cortex. The primary bone ranges from a fibrous to a pseudo-lamellar tissue (Francillon-Vieillot et al. 1990). Only the five most peripheral LAGs are preserved, being increasingly closer to each other (0.24–0.14 mm) as approaching the surface. The medullary cavity contains a few thick, irregularly aligned endosteo-endochondral trabeculae. Some remnants of globular calcified cartilage are present within the trabeculae. In the cortex, primary bone bears numerous, evenly-distributed osteocyte lacunae. They range from globular to flattened (8 μm long, 4 μm wide). In the secondary bone deposit, they are fewer, more flattened, and larger (12 μm), aligned in parallel to the secondary-bone surface.

#### Femur SMNS 81445—Kupferzell Locality

This section is again drop-shaped and completely preserved (Fig. 4b). The vascular network of the cortex falls into two kinds of textures: (1) the inner half of the cortex with numerous vascular lacunae, largely eroded to form large bays of erosion that are progressively covered with a thin layer of secondary bone; (2) in the outer half of the cortex, the vascular mesh is formed by primary small canals (lumen diameters: 100 μm in average). The cortex as a whole is largely compact, measuring 2.4 mm. It is fibro-lamellar in the inner half (fb, Fig. 4d) and consists of lamellar tissue in the outer half (lb, Fig. 4d). Some radial bundles of extrinsic fibres are present in the peripheral compact area of the mid-shaft section. Six LAGs were identified (black arrows, Fig. 4e), which are ever more closely set towards the periphery (0.63–0.19 mm). The medullary cavity is fully preserved, and with well-established cortex margin. In addition, remnants of calcified cartilage (cc, Fig. 4c) persist between the cortex and endosteal bone. The medullary cavity is crossed by several endosteo-endochondral trabeculae (eet, Fig. 4c). The osteocyte lacunae are numerous, evenly distributed and oriented in all directions in the primary tissue. Their size matches that of the lacunae in 81460. However, their outline is somewhat more flattened in the periphery. In the secondary bone tissue, shape and arrangement of osteocytes also match those in 81460.

#### Femur SMNS 91546—Kupferzell Locality

This drop-shaped section is also nicely 3D preserved. The distinction between the spongiosa and the compacta becomes clearer as the bays of erosion forming the spongiosa are increasing. On the contrary, the successive rows of vascular canals within the compacta are only slightly eroded (averaging diameter: 110 μm). The compact cortical thickness is constant, measuring 1.7 mm all over the section. It is mainly lamellar despite the fibro-lamellar component in its inner part. Some LAGs can be recognized but the whole LAG pattern cannot be followed all over the section. The spongiosa trabeculae are fully ossified and most of them are covered with a thin layer of endosteal bone. The osteocyte lacunae are numerous, evenly distributed and oriented in all directions in the primary tissue. They are round in the primary bone of the spongiosa (7 μm) and become slightly more flattened and larger in the compacta (10 μm long). In the endosteal bone, the osteocytes are very flattened with an average length of 20 μm.

#### Femur SMNS 81468—Vellberg Locality

This drop-shaped section is 3D preserved. There is a clear-cut limit between the compacta and the spongiosa. The general view of this section greatly reminds the section made in the femur SMNS 91546 from Kupferzell. The erosion has reached the exact same point. The LAG pattern is however more visible and exhibits six LAGs whose pattern narrows while the LAGs get closer to the external surface (distance of 30 μm between the most peripheral LAGs). Contrary to the observations made in the section SMNS 91546, a few remnants of calcified cartilage are still present within the spongiosa.

#### Femur SMNS 91544—Vellberg Locality

The thin section is crushed but the preservation of the femoral crest suggests that it also had a drop shape. Its greatly-eroded spongiosa results in the same organisation as the section SMNS 91548 from Kupferzell.

#### Femur SMNS 91548—Kupferzell Locality

The section has a drop shape and has remained very well preserved despite erosion spreading over the surrounding compact cortical bone tissue. The thickness of the compact bone is therefore very thin and irregular, ranging between 0.8 and 1.8 mm. The inner half of the compact cortical bone is pierced of rows of longitudinal vascular canals, already gathering to form bays of erosion due to intense erosion, while the outermost half is free of vascular canals. The three outermost LAGs are well recognisable and distant of 0.25 mm from each other. The remaining compact cortical bone is lamellar. The spongiosa is made of very thin bony trabeculae oriented in all directions and connecting successive concentric rings of bone. The centre of the spongiosa is very loose. A few remnants of calcified cartilage are still confined within spongiosa trabeculae. Most of the trabeculae are covered with endosteal bone. The osteocytes associated to the primary lamellar bone are flattened and measure 12 μm. The osteocytes within the secondary lamellar bone are 21 μm long. The cells in both types of bone are parallel to the lamellae of collagen fibres embedded in the bone matrix.

### Histotype B (Figs. 1, 5)

#### Femur SMNS 81440—Kupferzell Locality

**Fig. 5:**
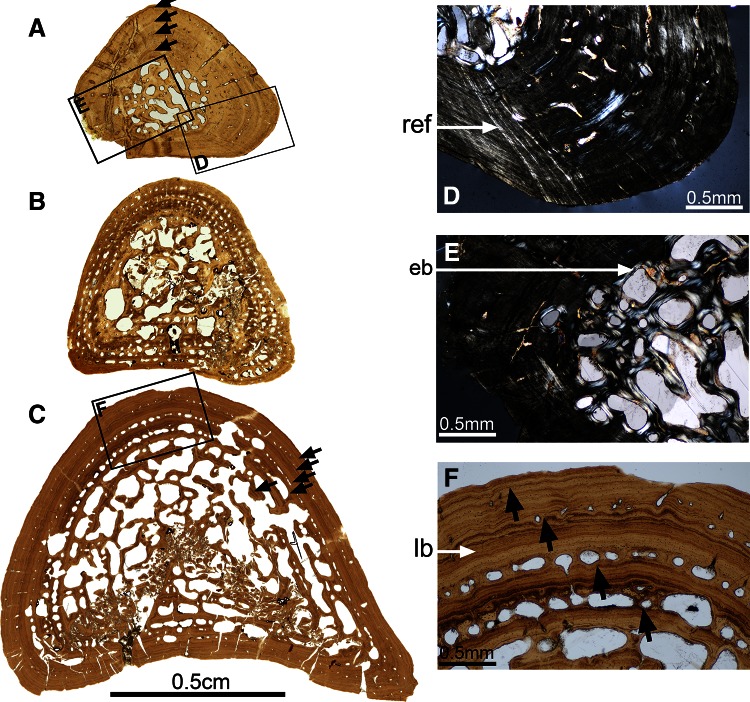
Histotype B—femur, *Gerrothorax pulcherrimus*. Mid-shaft transversal thin sections made in the femora 81440 (**a**), 81485 (**b**) and 81475 (**c**) show the impressive erosion of the cortex during the development. In the earliest stage, the cortex is very compact, made of fibrous or pseudo-lamellar bone, occasionally crossed by some radial extrinsic fibres (ref, **d**). Later in development, this cortex is pierced in its innermost region by numerous bays of erosion and become lamellar in the periphery (lb, **f**). The trabeculae in the medullary cavity are first made of endochondral bone (eb, **e**) in the earlier stages before their intense remodelling during the development of the femur. The lines of arrested growth can be followed by retrocalculation (Castanet et al. 2003), thereby permitting us to determine the developmental stage and the age of each specimen (*black arrows*, **a**, **c**, **f**). Photos **a**, **b**, **c**, **f** taken under natural light; photos **d**, **e** taken under polarized light

Almost the entire bone is preserved and of triangular outline (Fig. 5a). It is very compact, mostly pseudo-lamellar but slightly more fibrous in some areas. The cortical bone is 1.2 mm thick. Four LAGs were identified (Fig. 5a), with a distance of 0.40 mm to each other. Some radial bundles of extrinsic fibres (ref, Fig. 5d) span the entire width of the cortex. The vascular mesh contains very small primary canals and osteons (lumen diameters: 30 μm). Their alignment is mostly longitudinal but radial in some cases. The medullary cavity is bridged by several thick endosteal trabeculae (eb, Fig. 5b), which are oriented in a random fashion. There is no evidence of calcified cartilage. The erosion process had begun to extend into the innermost area of the cortex, forming several bays of erosion. These bays are covered with a 5–10-micron lamellar secondary bone deposit. In the primary bone tissue, osteocyte lacunae are numerous and mostly globular (10 μm). They are distributed evenly and isotropically. In the secondary bone deposit, they are very few, flattened (17 μm long, 4 μm wide) and all aligned to the secondary bone surface.

#### Femur SMNS 81485—Kupferzell Locality

This section is triangular and crushed in its central region and along one flank (Fig. 5b). The cortex is strongly eroded in the inner half, with large, irregular bays of erosion. The peripheral half is traversed by many longitudinal primary osteons. The diameters of the osteonal lumina range between 70 and 110 μm. Five LAGs were identified, at distances between 0.43 mm (inner part) and 0.25 mm from each other (periphery). The medullary cavity is difficult to distinguish from the cortex due to extensive erosion. The continuity between the cortical bony trabeculae (formed between the bays of erosion) and the endosteo-endochondral trabeculae in the medullary cavity precludes a distinction between cortex and medullary cavity. There are no remnants of calcified cartilage. The cortical bone is made of pseudo-lamellar and fibrous bone. The primary bone tissue contains numerous osteocytic lacunae, which are round in the inner half of the section and more flattened in the periphery. They are evenly distributed and oriented in all directions except in some small regions of pseudo-lamellar bone. Osteocytes measure 10 μm (round) in the primary bone. They are 20 μm long and 10 μm wide (flattened) in the secondary bone.

#### Femur SMNS 81496—Kupferzell Locality

This section is triangular, very spongy and badly crushed in the central region. The spongiosa (secondary) extends across most of the section. The trabeculae are very thin in the medullary cavity, but the margin of the medullary cavity is difficult to trace. Space between the trabeculae decreases towards the periphery of the cortex. The trabeculae of the spongy core have no preferential orientation. Only the most external 0.5 mm of cortical bone remains unaffected by the internal erosion process. Internal to this peripheral area, there is a concentric layer of longitudinal primary osteons (lumen diameters: 105 μm). In places, two osteons merge into a single lacuna. The primary cortical bone is fibrous in most of the cortex and becomes (pseudo-) lamellar in the outermost quarter. Only two LAGs were identified in the periphery, which are closely set (0.27 mm). In the primary bone, the cell lacunae are mainly small and rounded (10 μm), numerous and homogeneously distributed. They are slightly more flattened in the periphery and aligned in parallel to lamellar bone when present. The secondary bone deposit is very thin and few, large, and flattened cell lacunae are present in this secondary tissue (30 μm long, 10 μm wide).

#### Femur SMNS 81475—Kupferzell Locality

The section is triangular, but tip of the femoral crest is sharper than the others (Fig. 5c). The midshaft section is deeply remodelled. Only a thin peripheral layer (0.7 mm) of compact bone surrounds the spongy core. The spongy core exhibits a homogenous structure. The trabeculae are covered with a thin deposit of secondary bone. They are oriented in all directions. The peripheral compact bone contains two rows of osteons in the inner part. Very few vascular canals are present in the peripheral half, forming small primary canals (lumen diameters: 30–40 μm by average, maximally 60 μm). They are oriented longitudinally except near the two sharpest tips where the canals, associated with extrinsic fibres, are radially oriented. The peripheral compact bone is lamellar (lb, Fig. 5f). Five LAGs were identified (Fig. 5c, f), separated by 0.25 mm. In the primary bone deposit, the osteocyte lacunae are globular in the central region and slightly flattened in the periphery (12 μm). In the secondary bone tissue, they are larger (19 μm) and parallel the bone surface.

### Histotype C (Figs. 1, 6)

#### Femur SMNS 81478—Kupferzell Locality

**Fig. 6:**
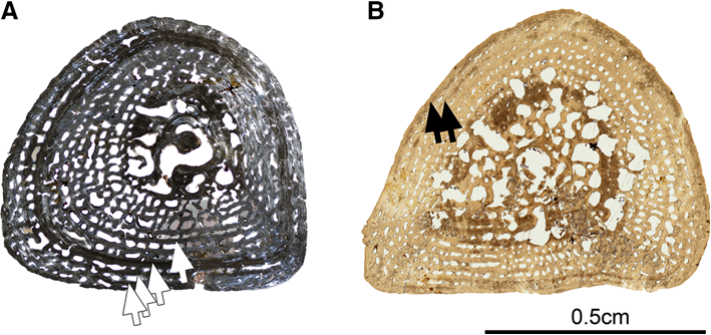
Histotype C—femur, *Gerrothorax pulcherrimus*. Mid-shaft transversal thin sections made in the femora 81478 (**a**) and 81466 (**b**) show the development of the central spongy core (made of endosteo-periosteal and endosteo-endochondral trabeculae) surrounded by some highly vascularised periosteal bone. Some lines of arrested growth (*arrows*) can be counted in both sections. Photo **a** taken under polarized light; photos **b** taken under natural light

The whole section is perfectly preserved and of slightly triangular outline (Fig. 6a). Some remnants of the medullary margins are preserved. The medulla is fully remodelled, precluding assessment of its size. The medullary cavity is crossed by several thick secondary-remodelled trabeculae. The cortex is relatively spongy, consisting of fibro-lamellar and pseudo-lamellar tissues. It houses eight successive concentric rows of osteons. The lumen diameters of the osteons usually range around 100 μm but may vary between 30 and 200 μm. Some osteons merge into larger lacunae. The cortex is 2.8 mm thick, crossed by bundles of extrinsic fibres in several areas. Near the femoral crest, osteons associated with extrinsic fibres are radial, contrasting with longitudinal osteons in the rest of the cortex. There are four obvious LAGs (white arrows, Fig. 5a). The distance between the innermost LAGs is 0.56 mm and the peripheral ones 0.24 mm. In the primary bone tissue, osteocyte lacunae are globular (8 μm), with a few being slightly flattened. They are homogeneously distributed and oriented in all directions. In the secondary bone tissue, the lacunae are very few, flattened, parallel the surface and match the size of those in the primary bone (14 μm).

#### Femur SMNS 81466—Kupferzell Locality

This section is mostly well preserved with a small crushed part only (Fig. 6b). The whole section is equally split into two areas: (1) a central, deeply eroded tissue (including the medullary cavity and the innermost region of the cortex) and (2) a substantially vascularised cortical area in periphery. The spongy core is affected by erosion that formed bays and endosteo-periosteal trabeculae that continue the endosteo-endochondral trabeculae of the medullary cavity. All these trabeculae are therefore covered with a layer of secondary bone deposit. The margin between the medulla and the cortex is not obvious. The peripheral cortical bone is traversed by at least five successive rows of longitudinal primary osteons (lumen diameters: 70–90 μm). The bone matrix is mostly pseudo-lamellar, but fibrous types are also established in some regions. Several LAGs were identified (black arrows, Fig. 6b), with the peripheral ones (0.2 mm distance) placed between successive osteon zones. In the primary bone matrix, osteocyte lacunae are numerous, mostly round, and arranged in all directions (10 μm). In the endosteal bone deposit, they are less numerous, more flattened (20 μm long, 10 μm wide) and aligned in parallel to the inner surface of the vascular lumina.

#### Femur SMNS 91547—Kupferzell Locality

This drop-shaped section is almost completely preserved. It shows a medullary cavity distinct from the cortex (1–2 mm). The primary cortical bone is pierced with vascular canals which have been significantly eroded, thereby forming a very spongy cortex (eroded lumen diameter: 135 μm on average). In the innermost cortex, several bays of erosion have appeared from secondary erosion of vascular canals merging into a large single bay. The resulting lacunae are then covered with a thin layer of secondary lamellar bone. The eroded vascular canals are longitudinally oriented and arranged circularly in the cortical bone. In the medullary cavity, the trabeculae are relatively thick and covered with a thin layer of endosteal bone. They are randomly arranged. Four LAGs are visible and distant of 0.3 mm. The primary bone is a fibro-lamellar matrix. There is no remnant of calcified cartilage. The bone cells are round and measure 7 μm on average in the primary bone. They are flattened, parallel to the lamellar bone and measure 13 μm long on average in the secondary bone.

### Histotype D (Figs. 1, 7)

#### Femur SMNS 91545—Vellberg Locality

**Fig. 7:**
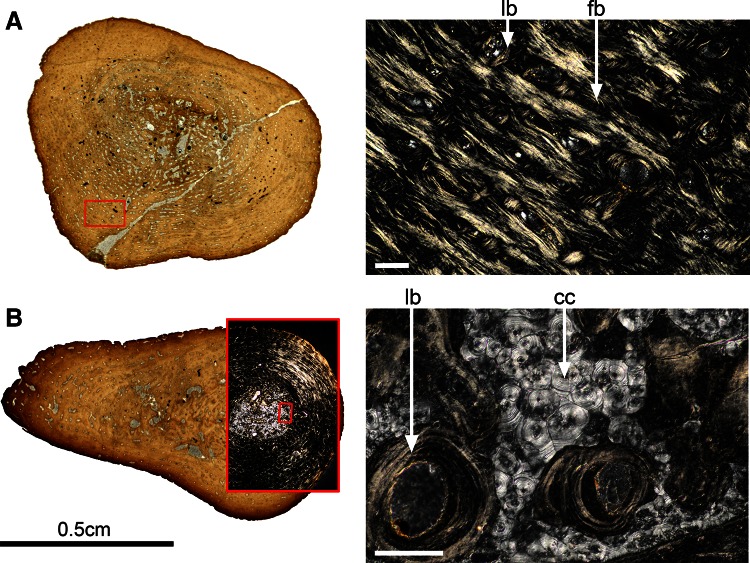
Histotype D—femur, *Gerrothorax pulcherrimus*. Mid-shaft transversal thin sections made in the femora 91545 (**a**) and 91543 (**b**) show thick and compact cortices. They are mostly made of fibro-lamellar bone, i.e. fibrous primary bone (fb, **a**) pierced with primary osteons exhibiting a layer of secondary lamellar bone (lb, **a**). The medullary cavity of the specimen 91543 is completely filled in with calcified cartilage (cc, **b**) surrounding lamellar endosteal trabeculae (lb, **b**). Low-resolution photos taken under natural light; detailed photos taken under polarized light. *Scale bars* for both detailed photos: 100 μm

This section is grossly round, slightly triangular (Fig. 7a). It is preserved in 3D. It is very compact (compactness: 0.943). The medullary cavity is obviously small and crossed by numerous thick bony trabeculae. The very large amount of cortical bone deposit (3 mm) is compact despite a dense vascular mesh constituted of longitudinal and circular small primary osteons (lumen diameters measure 40 μm on average), aligned in concentric rows. A few simple vascular canals can also be noticed. The primary bone ranges from a fibrous to a pseudo-lamellar tissue. There is no clear LAG pattern. In the cortex, osteocyte lacunae are small and evenly distributed within the primary bone. They are mainly globular (5 μm).

#### Femur SMNS 91543—Vellberg Locality

This section is triangular and elongated; it is fully preserved in 3D (Fig. 7b). Despite localized events of erosion, the thick cortex remains very compact (2 mm minimum; compactness: 0.915). The vascular network embedded in the cortex is made of a multitude of small longitudinal and circular primary osteons (lumen diameter: 35 μm on average). The cortex is mainly pseudo-lamellar, and fibro-lamellar in certain regions (Fig. 7b). Three LAGs, distant of 0.55 mm from each other, were identified. The osteocyte lacunae are sparse and evenly distributed in the primary tissue. Their shape and arrangement match those in SMNS 91545. The medullary cavity is fully preserved. It is completely filled in with bony lamellar trabeculae embedded in a matrix of calcified cartilage (Fig. 7b).

### Limb-Bone Histology of *Plagiosuchus pustuliferus*

**Fig. 8:**
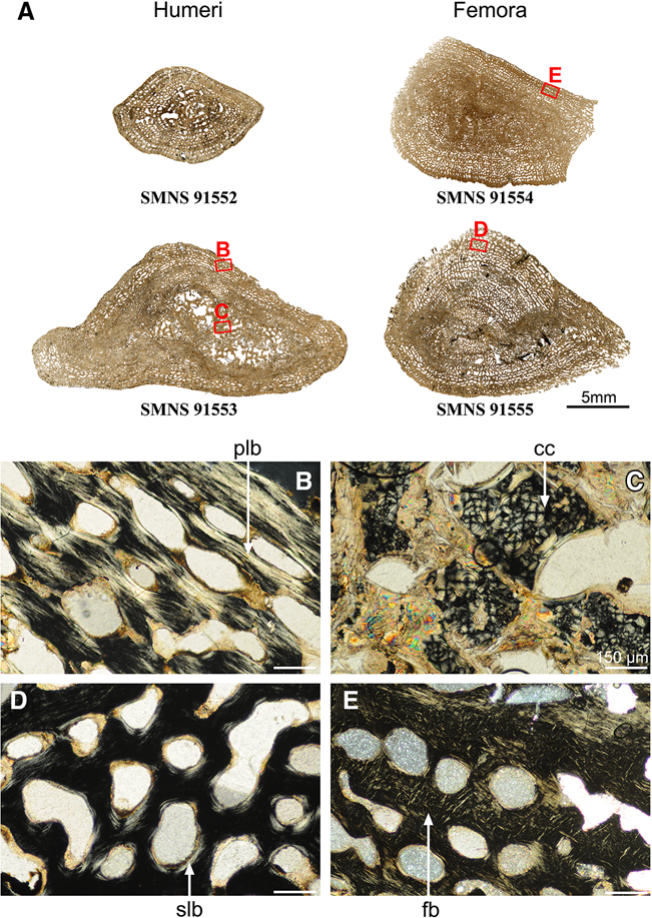
Histology of *Plagiosuchus pustuliferus*. Mid-shaft transversal thin sections made in two humeri and two femora of *Plagiosuchus pustuliferus* (**a**) show very spongy sections formed by the erosion of the numerous vascular canals of the cortical bone. These erosion lacunae are progressively covered with a thin layer of secondary lamellar bone (slb, **d**). The primary cortical bone evolves from a fibrous matrix (fb, **e**) to a pseudo-lamellar matrix (plb, **b**). In the medullary cavity, some remnants of calcified cartilage (cc, **c**) are still present, even late in development. *Scale bar* for the images **b, d, e**: 150 μm. Photo **a** taken under natural light; photos **b, c, d, e** taken under polarized light

Although the LAG pattern is not very much preserved, the different bone mid-shaft sizes suggest different developmental stages. Whatever the bone (femur or humerus) and the developmental stage, the mid-shaft histology of *Plagiosuchus pustuliferus* exhibits a very spongy pattern with no remaining compact cortical tissue (Fig. 8a). The spongiosa is formed by the endosteo-endochondral trabeculae within the medullary cavity and the erosion of the dense, regular vascular mesh from the cortical deposit. The primary tissue in the cortex is fibrous during the early development of the animal (Fig. 8e) and then turns pseudo-lamellar (Fig. 8b). The erosion bays are progressively covered with a thin layer of secondary bone deposit (Fig. 8d). Some remnants of calcified cartilage are still visible in the large specimens (Fig. 8c).

## Discussion

Understanding the histodiversity within vertebrates has long been of great interest for biologists (Castanet et al. 2001; Ricqlès et al. 2004). Amprino (1947) first proposed a causal explanation of bone histodiversity, suggesting that histological microstructural organisation would reflect growth rates. Since this discovery, quantitative analyses have proved “Amprino’s rule” to be exact (Castanet 1985; Castanet et al. 1996, 2000). We know today that the histodiversity of primary bone tissues (organisation of the bone matrix, size of bone-cell lacunae, density of the vascular mesh, thickness of the growth marks) reflects variations linked to speed differences of osteogenesis (Castanet et al. 1993; Ricqlès et al. 1991). These differences are usually related to intrinsic (metabolism, ontogeny, morphogenesis, phylogenetic heritage) or extrinsic factors (environmental constraints) (Castanet et al. 2001). Bone remodelling (i.e., erosion and secondary bone deposition) can also be controlled by epigenetic constraints (biomechanics, metabolisms, environment) (Castanet et al. 2001).

For instance, little-vascularised lamellar bone tissue with numerous close growth marks, usually reflects a slow osteogenesis (Ricqlès 1975; Castanet et al. 2001) while fibro-lamellar bone associated to a dense vascular mesh constituted of primary osteons would rather be the result of a rapid and regular osteogenesis (Ricqlès 1975; Castanet et al. 2001). Based on well-established relationships between skeletochronological/histological studies and intrinsic/extrinsic factors made on extant bone material, we propose here to understand the origin of the histodiversity (i.e., the diversity of histotypes) and discuss it within *G.* *pulcherrimus* from both Triassic localities of Kupferzell and Vellberg in Germany.

In order to affirm the existence of histodiversity (and therefore the existence of plasticity) within this species, similar palaeohistological observations have been made with another taxon from the Kupferzell locality: *P. pustuliferus*. This latter shows similar compactness, remodelling, vascularisation and bone matrix patterns whatever the limb bone and the developmental stage. By comparison with similar studies made on other Triassic temnospondyls from different Laurasian localities (Konietzko-Meier and Klein 2013; Steyer et al. 2004), *P. pustuliferus* can therefore be considered as representative of the standardized reaction norm, thereby suggesting that *G. pulcherrimus* exhibits an unusual great histodiversity.

### Developmental Plasticity

The limb-bone growth of most tetrapods is cyclical; the bone record at midshaft is therefore crossed by a series of circular growth marks (Castanet et al. 1993). Previous skeletochronological studies (Castanet et al. 1993, Castanet et al. 2003) have shown that each growth mark is punctuated by a line of arrested growth (LAG) and that such growth marks are usually formed annually (Castanet 1985; Castanet et al. 1993). This permits the inference of age data by counting the number of growth marks present in a limb-bone section at mid-shaft (Castanet et al. 1993, 2003; Castanet 1994). These data are not only informative about the somatic age, but the pattern of growth marks can also be used to identify the ontogenetic stage (Castanet et al. 2003). Once an animal reaches sexual maturity (i.e., it becomes an adult), its growth rate decreases. The mid-shaft growth in limb bones proportionally decreases as well. This slow-down of the mid-shaft growth can thus be easily revealed in adult specimens by the tightening of the most peripheral LAGs (Castanet et al. 2003).

Based on these observations made among living tetrapods, it is reasonable to suspect that the mechanisms of limb-bone growth within fossil tetrapods were the same (Sanchez et al. 2010a, 2010b; Sander et al. 2006; Steyer et al. 2004). It is thus possible to apply a skeletochronological analysis to determine the ontogenetic stages of the different plagiosaurid bones studied here.

#### Histotype 1

SMNS 81425 includes seven LAGs which progressively close up towards the periphery. Because the distance between the three last LAGs drastically decreased compared to the distance between the innermost LAGs, this individual can be considered as an early-adult individual, with its sexual maturity reached around 5–6 years (Table 1). In the larger individual 81382, remodelling of the innermost part of the bone has overprinted the pre-existing LAG pattern. Although it remains impossible to count all LAGs, the most peripheral LAGs in 81382 are closer than the most peripheral LAGs in 81425, suggesting that this individual had reached the sexual maturity and was probably an adult. 91550 being even larger could also be considered as an adult.

**Table 1:** Developmental characteristics observed and deduced from the different histotypes

Histotype	Juvenile characteristics	Adult characteristics
1	No calcified cartilage at the late juvenile stage Little erosion of the cortical bone; more intense erosion of the medullary trabeculae Little secondary-bone deposition Duration: 5 years Annual primary-bone deposit: 0.41 mm Compactness: 0.863	No calcified cartilage Vascular canals slightly more eroded than at the juvenile stage and all covered with a thin layer of secondary bone Extension of bays of erosion Peripheral annual primary-bone deposit: 0.22 mm Compactness: 0.806
2	Calcified cartilage present in the two youngest juveniles and absent in the oldest one Intense erosion in the cortical and medullary areas Remodelling with secondary-bone deposition begins early in development Duration: 6 years Annual primary-bone deposit: 0.41 mm Compactness evolving from 0.758 to 0.737	Remnants of calcified cartilage enclosed by secondary deposition Intense erosion mainly weakening the spongiosa, which only slightly spreads All the trabecular surfaces are covered with endosteal bone Peripheral annual primary-bone deposit: 0.24 mm Compactness evolving up to 0.71
A	Duration: 3–4 years Annual primary-bone deposition: 0.63 mm	Presence of calcified cartilage Erosion begins late at the adult stage and only affects the innermost 3/4 of the section Peripheral annual primary-bone deposit: 0.19 mm Compactness evolving from 0.861 to 0.885
B	No calcified cartilage even in the youngest individual Significant erosion process beginning early and increasing during the development Remodelling begins early in development Duration: 5–6 years Annual primary-bone deposit: 0.41 mm Compactness: 0.918	No calcified cartilage Intense erosion process Laying down of a thin layer of secondary bone on all the eroded surfaces Peripheral annual primary-bone deposit: 0.22 mm Compactness decreasing up to 0.685
C	Duration: at least 3 years and certainly more Annual primary-bone deposit: 0.56 mm	No calcified cartilage Consistent erosion of the vascular canals but preservation of the medullary cavity up to late in development (medullary cavity only eroded in the oldest individual) Slight remodelling activity Peripheral annual primary-bone deposit: 0.25 mm Compactness slightly increasing up to 0.867
D	Duration: at least 3 years and certainly more Annual primary-bone deposit: 0.55 mm Compactness evolving from 0.943 to 0.915	

The compactness of each section was quantified using the software *Bone Profiler* (Girondot and Laurin 2003)

#### Histotype 2

Representing histotype 2, the humeral mid-shafts of three specimens (81423, 81403, 91551) show the relatively regular LAG-pattern typical of juvenile tetrapods (Castanet et al. 1993). In the smallest thin section (81423), four equivalent growth marks are present across the cortex. In the larger specimens (81401 and 83781), while the inner growth marks are obscured by remodelling, the four most peripheral ones are clearly visible. The distances between LAGs in the innermost part of the thin sections are the same as in the smallest specimen. The distance between the two most peripheral LAGs however is significantly smaller, meaning that these individuals certainly had just reached the adulthood. By retrocalculation (Castanet et al. 2003; Leclair and Castanet 1987), the specimen 81401 has two additional peripheral LAGs, indicating that the juvenile period did not exceed six years (Table 1).

#### Histotype A

The mid-shaft section of the smallest femur (81460) shows a LAG pattern (at least five LAGs) that progressively tightens towards the periphery of the cortex. Because of poor preservation, only the most peripheral LAGs are unequivocal. Tightening indicates a late-juvenile to early-adult stage. In SMNS 81445, the whole series of growth marks is preserved indicating that this six-year-old specimen was already adult. All the larger specimens (91546, 81468, 91544 and 91548), showing a tightened LAG pattern, could be identified as adults. The juvenile stage in this histotype can therefore be considered as relatively short (no longer than 3–4 years; Table 1).

#### Histotype B

The smallest specimen (81440) exhibits a regular LAG pattern typical of juveniles (Castanet et al. 1993). The three other individuals (81485, 81496, 81475) show a tightening of LAGs towards the periphery, suggesting that they must have reached the adult stage (or almost reached it, for 81485). SMNS 81440 has four LAGs. Even if the slightly larger SMNS 81485 is a bit crushed in its innermost region, based on size comparative assessments with SMNS 81440, we can estimate its number of LAGs around six. This indicates that the juvenile stage ended at around 6 years in the histotype B (Table 1). The largest specimen (81475) is twice the size of the smallest (81440), suggesting a relatively long lifespan.

#### Histotype C

The LAG patterns of this histotype are not easily recognized. The section 81478 preserves at least two LAGs in the innermost region of the cortex and two LAGs in the periphery. These observations suggest that (1) the juvenile period of this individual must have lasted around 3 years (Table 1); and (2) this individual must have died at an early stage of its adulthood. In specimen 81466 and 91547, only the most peripheral LAGs are preserved and similarly tightened as in SMNS 81478. Specimen 81466 and 91547 are therefore interpreted as adults.

#### Histotype D

The midshaft section of both specimens (91545 and 91543) are of about the same size and exhibit equally complete distant LAG patterns suggesting that they can be considered as belonging to juvenile individuals (Castanet et al. 1993). The juvenile stage for this histotype would therefore last at least 3 years.

#### Comparative Conclusion on Developmental Plasticity

Among extant tetrapods, differences in age of sexual maturity acquisition could result from sexual dimorphism (Castanet et al. 2003) or developmental divergences constrained by surrounding environmental conditions (Castanet et al. 1988, 2001). With the juvenile period lasting twice as long in some histotypes than in others, the divergence is unlikely to represent either insignificant individual variation or sexual dimorphism. It has never been observed among wild caudate populations that one gender could grow to adulthood in half the time required by the other one (Castanet et al. 2003). Instead, these divergent developmental rates suggest non-sexual variation of developmental traits.

### Metabolic Plasticity (Table 1)

#### Histotype 1

In this histotype, there was little remodelling of the original compact cortex during the juvenile period, contrasted by a more intense remodelling of the medullary cavity. This is confirmed by the absence of calcified cartilage in the late juvenile specimen 81425. Remodelling increased during growth. The fibrous matrix of the primary bone tissue and the presence of round cells with homogeneous, isotropic distribution suggest that bone deposition proceeded at a high rate. The annual bone deposition was 0.41 mm during the juvenile stage and 0.22 mm during the adult stage.

#### Histotype 2

Remodelling started only superficially, early in development, as suggested by the remnants of calcified cartilage persisting between the primary cortical bone and endosteal deposit. The remodelling activity occurred both in the medullary cavity and cortical compact bone. The erosion process, which intensified during development, took place in the periphery of vascular canals, resulting in a peculiar pattern of successive eroded rows. The juvenile period was slightly longer than in histotype 1. The fibrous matrix and the occurrence of round cells in the central section (twice the size of those in the periphery) indicate a relatively high juvenile growth rate. Annual bone depositions were however equivalent to those of histotype 1.

#### Comparative Conclusions on Humeral Histotypes

The growth rate (based on annual bone deposit measurements, matrix and cellular organization) was similar in both histotypes but the development (i.e., the establishment of adult features) was slightly slower in histotype 2. Despite a longer juvenile period, remodelling was more intense in this histotype. This indicates a more intense metabolic activity for histotype 2.

#### Histotype A

Only adults, aged 5 years at least, represent this histotype. The remodelling process was relatively weak. The medullary cavity remains distinguishable until late in development (up to the stage of 81468), still preserving remnants of calcified cartilage. Erosion in the cortical bone started late during the adult period and only affected the innermost three quarters of the cortex. The primary vascular network therefore remained untouched in the periphery. The cortical matrix, regionalized as well (fibrous bone associated with the denser eroded vascular mesh in the central part and pseudo-lamellar bone in the periphery), suggests a drastic slow-down of bone deposition after sexual maturity was attained. In the periphery, the alignment of the osteocyte lacunae to the pseudo-lamellar matrix and the LAG pattern are consistent with this drastic slow-down. Annual bone deposition was 0.63 mm in juveniles, contrasted by 0.19 mm in adults.

#### Histotype B

The sections of different ontogenetic stages have been analysed. The smallest section (81440) belongs to a juvenile aged 4 years. Most of its cortex is compact but the limit between the medullary cavity and the cortical bone had already disappeared, indicating that remodelling had already begun. The absence of calcified cartilage is consistent with this observation. Remodelling strongly increased during development. In the three other specimens, erosion started in the periphery of vascular canals, forming rows of erosion, then spreading from the central region toward the periphery to form large bays of erosion. A thin layer of compact bone nevertheless always persists in periphery of the section. From the LAG pattern, we estimate the annual rate of bone deposition as 0.41 mm in juveniles and 0.22 mm in adults. The globular shape, great density and homogeneous distribution of the osteocyte lacunae also suggest that the intense process of primary bone deposition did not slow down as drastically as in histotype A.

#### Histotype C

This histotype is only represented by three adult specimens. The erosion activity differs from both previously described femoral histotypes. Here, remodelling was distributed evenly across the cortex in development. The medullary cavity is fully remodelled. The rate of erosion is therefore considered as relatively high during early development. During the adult period, larger bays of erosion began to spread in the central area of the section, around the medullary cavity. Even if the erosion process started early in development (extension of the bays of erosion), the disturbance of primary tissues remained local. The fibrous bone matrix and globular osteocytic lacunae indicate a relatively fast deposition of primary tissue. This is confirmed by the LAG pattern, which suggests annual bone deposition around 0.56 mm in juveniles and 0.25 mm on average in adults.

#### Histotype D

While all other histotypes showed a tendency to erosion, this histotype exhibited a very compact cortical bone and a medullary cavity that could remain filled in with calcified cartilage. The fibro-lamellar matrix and the high bone growth rate (0.55 mm) suggest a fast growth during the juvenile stage. In addition to a thick cortical deposit and a high growth rate, the presence of calcified cartilage conserved within the whole medullary space can be interpreted as a pachy-osteosclerotic phenomenon (Ricqlès and Buffrénil 2001).

#### Comparative Conclusions on Femoral Histotypes

Four different combinations of growth rate and remodelling patterns (defined here as four “histotypes”) indicate the existence of four different modes of metabolic activities.

Although growth rates in histotypes A and C were both relatively high, the rates of erosion and remodelling differ. Histotype A presents a moderate pattern of erosion that begins late in development while the erosion process begins early in histotype C. However, the remodelling process in histotype C does not reach the intensity observed in histotype B. In the latter, the remodelling not only begins early in the development but also fully disturb the primary organization of the midshaft histology with great intensity. The matrix and bone cell patterns however suggest a lower growth rate of the primary tissue in this histotype. The juvenile period in histotype B lasted twice longer than that of histotype A. The determination of the developmental features in histotype C resembles more those of histotype B than A: i.e., no calcified cartilage at the adult stage, and, a juvenile stage longer than three years. Histotype D however shows a very slow metabolic activity (no cortical remodelling, slow endochondral activity) despite a high growth rate, which greatly reflects a pachy-osteosclerotic condition.

#### Synthesis on Metabolic Plasticity

Two histotypes have the same metabolic features: humeral histotype 2 and femoral histotype B both formed under a relatively high growth rate of primary bone deposition, an intense remodelling process that started early in development and overprinted the whole section, a peculiar pattern of erosion focused on successive rows of vascular canals, and a slow-down of cortical growth after 6 years. Other histotypes however seem too much different to be associated to each other, meaning that the histodiversity was particularly great in Kupferzell. Due to missing corresponding humeri/femora, it is highly probable that there may be even more histological variation in both studied localities that was not captured in the analysed sample.

### Discussion on Gerrothorax’s Histodiversity

Because bone remodelling is homogeneously distributed all over the mid-shaft thin sections, the histodiversity observed in *Gerrothorax* limb bones could not be the result of localized morphogenesis. The diversity of metabolisms and developmental rates from bone histological inferences observed within different populations of a same species could only illustrate different activity rhythms (e.g., due to temperature or anoxia, Ricqlès 1989), life conditions (e.g., nutrient availability, Hough et al. 1988) or ecological stress (e.g., dryness, Caetano and Castanet 1993; e.g., seasonality, Castanet et al. 2001) as seen in extant tetrapods.

The presence of limb bones with a pachy-osteosclerotic pattern (histotype D) suggests a totally different adaptive strategy from other specimens. Indeed, pachy-osteosclerotic animals have only been observed in shallow water, mostly confined to estuarine, inter-tidal or undertow zones (Buffrénil and Schoevaert 1989; Ricqlès and Buffrénil 2001). Specimens showing a reduction in bone mass however have proved to be capable of a better manoeuvrability (Webb and Skadsen 1979), which is in agreement with a more intense metabolic activity (illustrated by eroded histotypes).

The histodiversity, especially due to erosion processes, in Kupferzell is greater (3 femoral histotypes and 2 humeral histotypes) than in Vellberg (2 femoral histotypes and 1 humeral histotype), suggesting that specimens from Kupferzell would exhibit a higher metabolic activity and a broader reaction norm in Kupferzell than in Vellberg.

### Palaeoecology and Environments

Although the time span covered by Kupferzell and Vellberg localities is minimal (at maximum, a few ten thousand years), understanding the developmental and metabolic plasticity within populations in each locality and between both localities could help understanding *Gerrothorax* adaptive strategies.

The Kupferzell locality and fauna form the focus of ongoing studies, centred on sedimentology, facies analysis, faunal assemblage, and geochemistry of tetrapod teeth (Schoch and Tütken, work in progress). The green calcareous mudstone layer, from which some here-analysed material is derived, is characterized by large quantities of bones and few articulated skeletons. The bulk of the material stems from *Gerrothorax pulcherrimus*, accompanied by large bones and partial skulls of *Mastodonsaurus giganteus*. The calcareous mudstone formed in a lake basin at least 5 km in diameter (N–S), whose richness in ostracods indicates good aeration. Abundant oogonia further testify the presence of characeans (Urlichs 1982), which are known to flourish in clean, carbonaceous water with low or medium nutrient levels (John et al. 2002). They may play a role as pioneer-colonizers or ephemerals in newly-formed ponds (Groves and Bullock-Webster 1924).

Large quantities of bones from the green mudstones are reworked and were accumulated in unnatural amounts at the Kupferzell site (max. range of that area ~700,000 m^2^). Reworking indicates that the accumulation of bones may have been caused by subsequent sheet floods that transported the prefossilized bones into a depression where they were finally deposited and that the lake fell dry once or repeatedly. Skeletons of lake dwellers (*Gerrothorax*, *Mastodonsaurus*, bony fishes) were first pre-fossilized during the dry periods and then eroded by subsequent floods, which led to their accumulation in channels at the lake floor (Urlichs 1982). Conditions before and after drying and prefossilization must have been similar, as ostracod faunas and characeans indicate.

The paucity of taxa at Kupferzell lake contrasts with the situation in the coeval lake at Vellberg (a 16 × 6 km large water body, 18 km SE from Kupferzell). This suggests that living conditions were not optimal for most aquatic tetrapods at Lake Kupferzell. *Mastodonsaurus* was an almost ubiquitous taxon in the Lower Keuper, forming the top predator of its time and capable of invading many habitats (Schoch and Milner 2000). The most common ostracod at Kupferzell (*Darwinula* *liassica*) was a freshwater-dweller, as was the rarely found bivalve *Unionites brevis* (Urlichs 1982). However, these ostracod and bivalve taxa, and especially the brackish *Speluncella* spp., are also known from oligo- and miohaline lake deposits (Urlichs 1982). This means that fluctuations in salinity are probable in Kupferzell lake. Cyclical resorption events are even preserved in the external periosteal bone of the osteoderms of *Gerrothorax* (Witzmann and Soler-Gijón 2010), a feature interpreted as a physiological response to periodic changes in salinity (Meunier and Gayet 1992). Indeed, a great demineralisation in scales, vertebrae and some other bones is hypothesized to release a large quantity of calcium necessary for the bone transformation and adaptation to salinity changes in salmons, sea trouts and eels during spawning migrations (Kacem et al. 2000; Meunier and Gayet 1992). The fish remains include *Dipteronotus* and a range of other bony fishes typical of freshwater-oligohaline conditions during the Middle Triassic.

In sum, the Kupferzell lake may be characterized as having consisted of clear water, not rich in nutrients, not stagnating, and of weak brackish salinity. Water level and salinity were probably fluctuating, thereby increasing non-favourable conditions and pushing the animals to a more active behaviour for surviving (as suggested by bone mass decrease and higher developmental and metabolic plasticities; Table 2). The Vellberg lake however formed a more quiet environment where pachy-osteosclerotic bottom-dwellers (represented by the histotype D) could remain inactive for long periods in shallow-water environments. The lower histodiversity in this locality (Table 2) would be in favour of better life conditions. This hypothesis is also consistent with the much higher diversity of tetrapods in the Vellberg lake as compared to Kupferzell.

**Table 2:** Distribution of the histodiversity within the Kupferzell and Vellberg localities

	Kupferzell locality	Vellberg locality
Histotype 1	3	0
Histotype 2	5	2
Histotype A	4	2
Histotype B	4	0
Histotype C	3	0
Histotype D	0	2
Total	19	6

The numbers in the table indicate the number of studied specimens

### Discussion on the Evolution of *Gerrothorax pulcherrimus*

*Gerrothorax pulcherrimus* is found in many deposits within the higher Lower Keuper (a time-averaged time slice) and across the whole Lower and Middle Keuper sequence (a 35-My time span). This species therefore occurred in many different palaeo-ecosystems: from shallow marine, brackish marshes and large salt lakes, to freshwater lakes, ephemeral ponds, rivers, and abandoned channels (Schoch and Witzmann 2012; Seegis 1997). However throughout this geographic, ecological, and temporal range, the species existed without traceable morphological variation. Gross morphology forms a stark contrast to the micro-anatomical level: our histological data testify an unusual extent of developmental and metabolic plasticity in *G. pulcherrimus* that could finally only be explained by environmental constraining fluctuations. Evidently, this species was not only long-lived and ecologically flexible through geological time, but also able to cope with rapid changes in salinity and other environmental factors (Witzmann and Soler-Gijón 2010), such as in the preserved, rather short-timed sequence studied here. Although the specimens studied here only represent a maximum of a ten-thousand-year time-span, the environmental conditions have been very much divergent between both localities and greatly fluctuating in Kupferzell over several hundred years. Time scale would therefore be too short to reflect microevolutionary changes but appropriate to provide substantial explanations to *G. pulcherrimus*’s plasticity.

*Gerrothorax pulcherrimus* is likely to have formed a pioneer species that settled in inhospitable water bodies. This hypothesis thus explains its abundance in deposits with poor faunas, and its low frequency in richer habitats (whatever the locality: in Kupferzell lake and at the base of the lake horizon at Vellberg).

Despite its fully aquatic nature, which precluded emigration during droughts, *G. pulcherrimus* forms one of the most characteristic taxa in the European Middle and Upper Triassic. Analogous to the broad reaction norms of lissamphibians, this fully aquatic plagiosaurid was able to persist to some conditions too harsh for many other tetrapods (e.g., salinity fluctuations and low levels of nutrients at Kupferzell lake), thereby maintaining its stable morphology despite environmental changes; these were compensated by developmental and metabolic adjustments. These new findings add to the picture of broad reaction norms in temnospondyls, which have recently been proposed for some Carboniferous and Permian taxa (Schoch 2009a, b).

The example of *G. pulcherrimus*, if confirmed with new material from different time-period localities, could illustrate that its morphological stasis is not the result of a general homeostasis and certainly not related to a developmental, genetic and environmental stability. On the contrary, it seems that the environmental instability in the case of *G. pulcherrimus* could constrain the populations to maintain a certain apparent consistency through a drastic internal remodelling affecting the metabolic and developmental functions. The current observations of concomitant histological plasticity and morphological consistency within a single taxon would support the concept of ‘canalization’ first proposed by Waddington in 1942 (Debat and David 2001; Flatt 2005). A plausible explanation is thus that the remarkable morphological stasis of *Gerrothorax* would have resulted from the environmental canalization, made possible by a buffered phenotype against numerous environmental perturbations. Only the microanatomical and histological levels would have conserved their plastic ability to adapt to these fluctuations. Palaeohistological analyses could therefore shed new light on the evolutionary strategy of this species to respond by adjusting growth rate and timing of developmental events in fluctuating environmental conditions.

## Conclusion

*Gerrothorax pulcherrimus* was one of the few vertebrate taxa that existed from the Middle Triassic (in Europe dominated by archosauriforms and nothosaurs) well through the Late Triassic, when dinosaurs diversified on land, phytosaurs flourished in streams, and turtles and mammals originated. This time span was a period of harsh conditions in Central Europe, as evidenced by the low diversity of lake dwellers in most strata (Seegis 1997). Understanding the plausible evolutionary strategy of non-amniotic dwellers such as *Gerrothorax* based on the combined study of anatomy, histology, and palaeoecology is therefore crucial.

Not only informative for a better understanding of fossil palaeoecosystems, this combined methodological approach, applied on fossil vertebrates, is also very promising for widening the possibilities of elucidating variability mechanisms from the population scale (coming from known horizons) to the taxon scale (when the stratigraphic log is complete). This could help filling the gap between palaeobiologists and evolutionary biologists regarding their different points of view resulting from different temporal-scope references and methodologies employed (Bell 2000; Kemp 1999), in order to finally understand factors of variation in enigmatic evolutionary events such as stasis.

## References

[CR1] Alcobendas M, Castanet J (2000). Bone growth plasticity among populations of *Salamandra salamandra*: Interactions between internal and external factors. Herpetologica.

[CR2] Amprino R (1947). La structure du tissu osseux envisagée comme expression de différences dans la vitesse de l’accroissement. Archives de Biologie.

[CR3] Bell MA (2000). Bridging the gap between population biology and paleobiology. Evolution.

[CR4] Benton MJ (1995). Diversification and extinction in the history of life. Science.

[CR5] Caetano MH, Castanet J (1993). Variability and microevolutionary patterns in *Triturus marmoratus* from Portugal: Age, size, longevity and individual growth. Amphibia-Reptilia.

[CR6] Castanet J (1985). La squelettochronologie chez les Reptiles I. Résultats expérimentaux sur la signification des marques de croissance squelettiques chez les Lézards et les Tortues. Annales des Sciences Naturelles, Zoologie.

[CR7] Castanet J (1994). Age estimation and longevity in Reptiles. Gerontology.

[CR8] Castanet J, Baez M (1991). Adaptation and evolution in *Gallotia* lizards from the Canary Islands: Age, growth, maturity and longevity. Amphibia-Reptilia.

[CR9] Castanet J, Cubo J, de Margerie E (2001). Signification de l’histodiversité osseuse: le message de l’os. Biosystema.

[CR10] Castanet J, Francillon-Vieillot H, de Ricqlès A, Heatwole H, Davies M (2003). The skeletal histology of the amphibia. Amphibian biology vol. V-osteology.

[CR11] Castanet J, Francillon-Vieillot H, Meunier F-J, de Ricqlès A, Hall BK (1993). Bone and individual aging. Bone.

[CR12] Castanet J, Grandin A, Abourachid A, de Ricqlès A (1996). Expression de la dynamique de croissance dans la structure de l’os périostique chez *Anas platyrhynchos*. Compte-rendu de l’Académie des Sciences de Paris.

[CR13] Castanet J, Newman DG, Saint Girons H (1988). Skeletochronological data on the growth, age, and population structure of the Tuatara, *Sphenodon punctatus*, on Stephens and Lady Alice Islands, New Zealand. Herpetologica.

[CR14] Castanet J, Rogers KC, Cubo J, Boisard J–J (2000). Periosteal bone growth rates in extant ratites (ostriche and emu). Implications for assessing growth in dinosaurs. Compte-rendu de l’Académie des Sciences de Paris.

[CR15] Charlesworth B, Lande R, Slatkin M (1982). A neo-Darwinian commentary on macroevolution. Evolution.

[CR16] Cubo J, Ponton F, Laurin M, de Margerie E, Castanet J (2005). Phylogenetic signal in bone microstructure of Sauropsids. Systematic Biology.

[CR17] Damiani RJ, Schoch RR, Hellrung H, Werneburg R, Gastou S (2009). The plagiosaurid temnospondyl *Plagiosuchus pustuliferus* (Amphibia: Temnospondyli) from the Middle Triassic of Germany: Anatomy and functional morphology of the skull. Zoological Journal of the Linnean Society.

[CR18] de Buffrénil V, Schoevaert D (1989). Données quantitatives et observations histologiques sur la pachyostose du squelette du dugong, *Dugong dugong* (Müller) (Sirenia, Dugongidae). Canadian Journal of Zoology.

[CR19] de Ricqlès A (1975). Recherches paléohistologiques sur les os longs des tétrapodes. VII- Sur la classification, la signification fonctionnelle et l’histoire des tissus osseux de tétrapodes (Première partie: Structures). Annales de Paléontologie.

[CR20] de Ricqlès A (1989). Les mécanismes hétérochroniques dans le retour des tétrapodes au mileu aquatique. Geobios.

[CR21] de Ricqlès A, Castanet J, Francillon-Vieillot H (2004). The ‘message’ of bone tissue in paleoherpetology. Italian Journal of Zoology.

[CR22] de Ricqlès A, de Buffrénil V, Mazin J-M, de Buffrénil V (2001). Bone histology, heterochronies and the return of tetrapods to life in water: Where are we?. Secondary adaptation of tetrapods to life in water.

[CR23] de Ricqlès A, Meunier F-J, Castanet J, Francillon-Vieillot H, Hall BK (1991). Comparative microstructures of bone. Bone matrix and bone specific products.

[CR24] Debat V, Alibert P, David P, Paradis E, Auffray J-C (2000). Independence between developmental stability and canalization in the skull of the house mouse. Proceedings of the Royal Society of London B.

[CR25] Debat V, David P (2001). Mapping phenotypes: Canalization, plasticity and developmental stability. Trends in Ecology & Evolution.

[CR26] Eldredge N, Gould SJ, Schopf TJM (1972). Punctuated equilibria: An alternative to phyletic gradualism. Models in paleobiology.

[CR27] Eldredge N, Thompson JN, Brakefield PM, Gavrilets S, Jablonski D, Jackson JBC (2005). The dynamics of evolutionary stasis. Paleobiology.

[CR28] Flatt T (2005). The evolutionary genetics of canalization. The Quarterly Review of Biology.

[CR29] Francillon-Vieillot H, de Buffrénil V, Castanet J, Géraudie J, Meunier F-J, Sire J-Y, Carter JG (1990). Microstructure and mineralization of vertebrate skeletal tissues. Skeletal biomineralization: Patterns, processes and evolutionary trends., vol. volume I.

[CR30] Futuyma DJ (2010). Evolutionary constraint and ecological consequences. Evolution.

[CR31] Girondot M, Laurin M (2003). Bone profiler: A tool to quantify, model, and statistically compare bone-section compactness profiles. Journal of Vertebrate Paleontology.

[CR32] Gould SJ (2002). The structure of evolutionary theory.

[CR33] Groves J, Bullock-Webster GR (1924). The British Charophyta. Vol. 2, Characeae.

[CR34] Hansen TF, Houle D, Pigliucci M, Preston K (2004). Evolvability, stabilizing selection, and the problem of stasis. Phenotypic integration: Studying the ecology and evolution of complex phenotypes.

[CR35] Hellrung H (2003). Gerrothorax pustuloglomeratus, ein Temnospondyle (Amphibia) mit knöcherner Branchialkammer aus dem Unteren Keuper von Kupferzell (Süddeutschland). Stuttgarter Beiträge zur Naturkunde B.

[CR36] Hough S, Avioli LV, Muir H, Gelderblom D, Jenkins G, Kurasi H, Slatopolsky E, Bergfeld MA, Teitelbaum SL (1988). Effects of hypervitaminosis A on the bone and mineral metabolism of the rat. Endocrinology.

[CR37] Jenkins JFA, Shubin NH, Gatesy SM, Warren A (2008). *Gerrothorax pulcherrimus* from the upper Triassic Fleming Fjord formation of East Greenland and a reassessment of head lifting in Temnospondyl feeding. Journal of Vertebrate Paleontology.

[CR38] John DM, Whitton BA, Brook AJ (2002). The freshwater algal flora of the British Isles.

[CR39] Kacem A, Gustafsson S, Meunier FJ (2000). Demineralization of the vertebral skeleton in Atlantic salmon *Salmo salar* L. during spawning migration. Comparative Biochemistry and Physiology Part A.

[CR40] Kemp TS (1999). Fossils and evolution.

[CR41] Konietzko-Meier D, Klein N (2013). Unique growth pattern of *Metoposaurus diagnosticus**krasiejowensis* (Amphibia, Temnospondyli) from the upper Triassic of Krasiejów, Poland. Palaeogeography, Palaeoclimatology, Palaeoecology.

[CR42] Leclair R, Castanet J (1987). A skeletochronological assessement of age and growth in the frog *Rana pipiens* Schreber (Amphibia, Anura) from Southwestern Quebec. Copeia.

[CR43] Mayhew PJ, Jenkins GB, Benton TG (2008). A long-term association between global temperature and biodiversity, origination and extinction in the fossil record. Proceedings of the Royal Society of London B.

[CR44] McElwain JC, Punyasena SW (2007). Mass extinction events and the plant fossil record. Trends in Ecology & Evolution.

[CR45] Meunier FJ, Gayet M (1992). Nouveau remaniement de la ganoïne chez un Semionodontidae du Crétacé supérieur de Bolivie: Interprétations paléobiologiques. Geobios.

[CR46] Montes L, Le Roy N, Perret M, de Buffrénil V, Castanet J, Cubo J (2007). Relationships between bone growth rate, body mass and resting metabolic rate in growing amniotes: A phylogenetic approach. Biological Journal of the Linnean Society.

[CR47] Ruta M, Benton MJ (2008). Calibrated diversity, tree topology and the mother of mass extinctions: The lesson of temnospondyls. Palaeontology.

[CR48] Sanchez S, de Ricqlès A, Schoch RR, Steyer J-S (2010). Developmental plasticity of limb bone microstructural organization in *Apateon*: Histological evidence of paedomorphic conditions in branchiosaurs. Evolution & Development.

[CR49] Sanchez S, Schoch RR, de Ricqlès A, Steyer J-S, Vecoli M, Clement G, Meyer Berthaud B (2010). Palaeoecological and palaeoenvironmental influences revealed by long-bone palaeohistology: The example of the Permian branchiosaurid *Apateon*. The terrestrialization process: Modelling complex interactions at the biosphere–geosphere Interface.

[CR50] Sander PM, Mateus O, Laven T, Knötschke N (2006). Bone histology indicates insular dwarfism in a new Late Jurassic sauropod dinosaur. Nature.

[CR51] Schoch RR (2009). Life-cycle evolution as response to diverse lake habitats in Paleozoic amphibians. Evolution.

[CR52] Schoch RR (2009). Evolution of life cycles in early amphibians. Annual Review of Earth and Planetary Science.

[CR53] Schoch RR, Milner AR (2000). Stereospondyli: Stem-Stereospondyli, Rhinesuchidae, Rhytidostea, Trematosauroidea, Capitosauroidea.

[CR54] Schoch RR, Witzmann F (2011). Bystrow’s paradox—gills, fossils, and the fish-to-tetrapod transition. Acta Zoologica.

[CR55] Schoch RR, Witzmann F (2012). Cranial morphology of the plagiosaurid *Gerrothorax pulcherrimus* as an extreme example of evolutionary stasis. Lethaia.

[CR56] Seegis D (1997). Die Lehrbergschichten im Mittleren Keuper von Süddeutschland—Stratigraphie, Petrographie, Paläontologie, Genese.

[CR57] Smith RHM, Ward PD (2001). Pattern of vertebrate extinctions across an event bed at the Permian-Triassic boundary in the Karoo Basin of South Africa. Geology.

[CR58] Stenseth NC, Maynard Smith J (1984). Coevolution in Ecosystems: Red queen evolution or stasis?. Evolution.

[CR59] Steyer J-S, Laurin M, Castanet J, de Ricqlès A (2004). First histological and skeletochronological data on temnospondyl growth: Palaeoecological and palaeoclimatological implications. Palaeogeography, Palaeoclimatology, Palaeoecology.

[CR60] Tanner LH, Lucas SG, Chapman MG (2004). Assessing the record and causes of late Triassic extinctions. Earth-Science Reviews.

[CR61] Thomas CD, Cameron A, Green RE, Bakkenes M, Beaumont LJ, Collingham YC (2004). Extinction risk from climate change. Nature.

[CR62] Urlichs M (1982). Zur Stratigraphie und Fossilführung des Lettenkeupers (Ob. Trias) bei Schwäbisch Hall (Baden-Württemberg). Jahresberichte des oberrheinischen geologischen Vereins, Neue Folge.

[CR63] van Bocxlaer B, van Damme D, Feibel CS (2008). Gradual versus punctuated equilibrium evolution in the Turkana Basin molluscs: Evolutionary events or biological invasions?. Evolution.

[CR64] Waddington CH (1942). Canalization of development and the inheritance of acquired characters. Nature.

[CR65] Waddington CH (1957). The strategy of the genes: A discussion of some aspects of theoretical biology.

[CR66] Webb P, Skadsen JM (1979). Reduced skin mass: An adaptation for accelaration in some teleost fishes. Canadian Journal of Zoology.

[CR67] Wild R (1980). The fossil deposits of Kupferzell, southern Germany. Mesozoic Vertebrate Life.

[CR68] Witzmann F, Soler-Gijón R (2010). The bone histology of osteoderms in temnospondyl amphibians and in the chroniosuchian *Bystrowiella*. Acta Zoologica.

